# P59 Focus on enmetazobactam chemical stability in association with cefepime for prolonged infusions, in syringe and elastomeric diffusers

**DOI:** 10.1093/jacamr/dlaf118.066

**Published:** 2025-07-14

**Authors:** Jon Ward, Chayem Akim, Juan Quevedo, Noëlle Jemmely, Elise D´Huart

**Affiliations:** Advanz Pharma UK, London, UK; Pharmacy Dept., CHRU Nancy, France; Advanz Pharma UK, London, UK; Advanz Pharma UK, London, UK; Pharmacy Dept., CHRU Nancy, France

## Abstract

**Background:**

Enmetazobactam, a N-methylated tazobactam derivative is a novel extended-spectrum β-lactamase inhibitor with a unique mechanism that overcomes tazobactam-resistant variants of class A β-lactamases, and is the first β-lactamase inhibitor approved in combination with cefepime in both the US and Europe with recently completed clinical trial. There is much research interest in prolonged and continuous infusions (collectively, extended infusions) of β-lactams to improve patient outcomes, particularly in critically ill patients in intensive care. Extended infusion of β-lactam antibiotics may offer clinical benefits aligned with improved probability of target attainment for critical pharmacokinetic/pharmacodynamic parameters that correlate with efficacy.

**Methods:**

To study the chemical stability of cefepime-enmetazobactam (FEP/META) in polypropylene syringe, silicone and polyisoprene elastomeric diffusers at different times using different solvents (NaCl 0.9% (NS) or Dextrose 5% (D5W)) and conditions (Syringes: 22-25°C, light exposition:T0h, T8h, T12h and T24h; Elastomeric devices: 32°C, no light exposition: T0h, T8h, T12h and T24h) through HPLC method and pH measurements. The studied concentration of FEP/META was 125/31.25 mg/mL in syringes (Q.S.F 48 mL) and 25/6.25 mg/mL in elastomeric devices (Q.S.F 240 mL). Enmetazobactam is considered chemically stable if it retains more than 90% of its initial concentration (Ci), with a maximum of variation of 1 pH unit.

**Results:**

Under all the conditions tested, for each container, for each concentration evaluated and for the two solvents used, enmetazobactam retained more than 90% of the Ci for 24 h. Under all conditions tested, the pH variation was less than 1 pH unit, which is within the acceptance limit.

**Conclusions:**

These promising results show that enmetazobactam has excellent chemical stability at all conditions and seems to be suitable for use in extended infusions in different medical devices. This might be an aspect to consider when using it in combination with cefepime with the aim to achieve a better probability of target attainment and improve patient outcomes, particularly in critically ill patients in intensive care.

